# Risk Assessment of Hepatocellular Carcinoma with Aflatoxin B_1_ Exposure in Edible Oils

**DOI:** 10.3390/toxins14080547

**Published:** 2022-08-11

**Authors:** Farhat Jubeen, Nida Zahra, Zill-i-Huma Nazli, Muhammad K. Saleemi, Farheen Aslam, Iram Naz, Lamia B. Farhat, Asmaa Saleh, Samar Z. Alshawwa, Munawar Iqbal

**Affiliations:** 1 Department of Chemistry, Government College Women University, Faisalabad 38000, Pakistan; 2 Department of Pathology, University of Agriculture, Faisalabad 38040, Pakistan; 3 Department of Biotechnology, Lahore College for Women University, Lahore 54000, Pakistan; 4 Department of Chemistry, College of Sciences, King Khalid University, P.O. Box 9004, Abha 61413, Saudi Arabia; 5 Laboratoire des Matériaux et de L’Environnement Pour le Développement Durable LR18ES10, 9 Avenue Dr. Zoheir Sai, Tunis 1006, Tunisia; 6 Department of Pharmaceutical Sciences, College of Pharmacy, Princess Nourah bint Abdulrahman University, P.O. Box 84428, Riyadh 11671, Saudi Arabia; 7 Department of Chemistry, Division of Science and Technology, University of Education, Lahore 54770, Pakistan

**Keywords:** AFB_1_, AFs, HPLC, dietary intake, MOE, quantitative liver cancer, risk assessment, Faisalabad

## Abstract

Contamination of edible oils with aflatoxins (AFs) is a universal issue due to the detrimental effects of aflatoxins on human health and the fact that edible oils are a major source of fungal growth, particularly storage fungi (*Aspergillus* sp.). The objective of this study was to assess aflatoxin B_1_ (AFB_1_) in edible oil used in fried food in order to determine the risk of cancer from AFB_1_ exposure through cooked food using the FAO/WHO’s and EFSA’s margin of exposure (MOE) quantitative liver cancer risk approaches. Using Mycosep 226 columns and HPLC-FLD, 100 samples of cooking oils (soybean, canola, and sunflower oil) from different food points were analyzed for contamination with aflatoxins. Of all the samples tested, 89% were positive for total aflatoxins and AFB_1_, with 65% indicating AF concentrations beyond permitted levels. Canola oil was found to contain higher levels of AFB_1_ and AFs than soybean and sunflower oil. Almost 71 percent of canola oil samples (range of 54.4–281.1 µg/kg) were contaminated with AF levels higher than the proposed limits of the European Union (20 µg/kg). The consumption of canola oil samples used in fried foods had MOE values that were significantly lower as compared to sunflower and soybean oils, indicating that risk reduction is feasible. Additionally, compared to soybean and sunflower oil, canola oil exhibited a greater threat of liver cancer cases linked to AFB_1_ exposure (17.13 per 100,000 males over 35 and 10.93 per 100,000 females over 35). Using a quantitative liver cancer approach, health risk valuation demonstrated that males and females over the age of 35 are at significant risk of developing liver cancer. The health risk assessment exposed that the males and female over the age of 35 are at considerable risk of liver cancer by using a quantitative liver cancer approach. The innovation of this study lies in the fact that no such study is reported related to liver cancer risk evaluation accompanied with AFB_1_ exposure from consumed edible oil. As a result, a national strategy must be developed to solve this problem so that edible oil products are subjected to severe regulatory examination.

## 1. Introduction

Edible cooking oils extracted from different seeds having a high content of natural antioxidants and unsaturated fatty acids are good for cardiac health, due to their long shelf-life [1]. Due to increasing health concerns in the twenty-first century, vegetable oils are favored for food frying or use in food processing industries over animal oils [2]. Consumers employ edible oil in their everyday routines because it avoids arteriosclerosis and lowers blood lipid levels [3,4]. The demand and consumption of edible cooking oils have increased globally, particularly in developed countries, as more consumers become aware of the health benefits of using it [4]. The most widely used oils in the world are peanut, maize, olive, canola, soybean, and sunflower oil, and their consumption is increasing. The Food and Agriculture Organization (FAO) estimates that 244.8 million tons of oil and fats were consumed worldwide in 2020–2021 [5]. The processing of oil, as well as production, packaging, transportation, and storage, may lead to AF contamination. In addition, advances in industrial processing, conventional agricultural methods, and climatic and pollution conditions such as: water, phosphorus, invisible insoluble compounds, free fatty acids, hexane, benzo pyrene, minerals, and pesticides; mycotoxins such as iron, lead, gossypol, and copper; and various primary and secondary oxidation products may result in the formation of novel harmful residues in edible oils [6,7]. 

Contamination of food and food commodities with aflatoxin (AF) is a potential hazard to global food. These Afs are human liver carcinogens that are toxigenic, carcinogenic, mutagenic, and teratogenic, causing serious health problems in humans and animals. They are lethal secondary metabolites fashioned by *Aspergillus flavus*, *A. prasiticus*, and *A. nomius*: the most toxic and active metabolite is AFB_1_ [8,9]. AFs predominantly damage the liver, but they might also impact other tissues and organs, such as the lungs and kidneys, to a lesser extent. Acute signs of aflatoxicosis in animals include bile duct development [10]. 

Pakistan is a tropical country; hence the climatic variables are likely to be favorable for the synthesis of fungal metabolites. Aflatoxigenic fungus thrives in environments with high temperatures and humidity levels. In several surveys conducted around the world, exposure to AFs in vegetable oils has been reported. In most cases, fungal contamination in edible cooking oils emerges as a consequence of moisture or temperature. Mold growth is fostered substantially by improper storage conditions and other eco-physiological factors, which are especially prevalent in the tropics and subtropics (where high temperatures and humidity prevail). However, AFs were found in high concentrations in animal feed as well as in food samples (cereal products) [11,12,13,14,15]. To the best of our knowledge, no prior research has been conducted in Pakistan regarding the risk assessment of liver carcinoma related to AFB_1_ in edible cooking oil.

Hepatocellular carcinoma (HCC) causes liver cancer and in developing countries is the third highest source of cancer deaths, with a 16–32 times greater risk of mortality [16]. About 25,200–155,000 new incidences of liver cancer are reported globally each year, which might be related to the consumption of peanuts and maize contaminated with AFs: people with HBV (hepatitis B virus) are particularly susceptible to this [17]. Conditions in Pakistan are far worse than those in Western Europe and North America, yet not as bad as that in Sub-Saharan Africa [18]. Today, reports and updated databases on fungal incidence and mycotoxin contaminations in marketed commodities are readily accessible. Health regulatory authorities have imposed rigorous regulations, notably for imported commodities [19,20]. In addition, aflatoxin B_1_ (AFB_1_) has a toxic effect on the liver. As a result, AFB_1_ has a substantial impact on the liver’s ability to metabolize lipids, carbohydrates, and proteins. The toxin hinders RNA polymerase and consequent synthesis of proteins at a higher rate in ducks than in rats, which is likely due to the fact that ducks have faster liver metabolism of AFB_1_ and mechanism for the same has been reported elsewhere [21]. 

Therefore, the purpose of this research was to estimate the level of aflatoxins (AFB_1_, AFB_2_, AFG_1_, and AFG_2_) in edible oils from various local restaurants throughout Punjab, Pakistan, as well as to perform a cancer risk evaluation of AFs owing to the use of these edible oils through risk characterization in males and females of different ages.

## 2. Results and Discussion

### 2.1. Screening of Edible Oil Samples for AF Contamination

As presented in Table 1, aflatoxin B_1_ (AFB_1_) and total aflatoxin (AF) contamination were in found in twenty-seven (87.9%) out of thirty-one samples of canola oil, twenty-two (61.1%) out of thirty-six samples of sunflower oil, and twenty (63.5%) out of thirty-two samples of soybean edible cooking oil (single and double frying oils). In canola oil, all single step frying oil samples were positive for AFB_1_ as well as AF contamination: 23.52% (4/17) were contaminated with total AFs below the permissible limits and 76.47% (13/17) showed AF levels above the permissible limits, whereas the two step frying oil samples showed 28.57% (4/14) below and 71.42% (10/14) above the permissible limits (20 μg/kg) set by the IAEA and WHO. The effect of single step and double step frying was not statistically significant for the incidence of total AFs, which can be justified by the fact that AFs are highly thermostable compounds: if an oil sample is contaminated with AFs, cooking practices are inadequate to degrade/remove contaminating AFs [22]. In sunflower oil, single step and double step frying samples showed 33.3% (6/18) and 44.44% (8/18) of AFs below and 66.67% (12/18) and 55.56% (10/18) of AFs above the permissible limits, respectively. An approximately similar incidence of AFs was observed in soybean oil, with the highest contamination of 69.23% (9/13) above the permissible limits in double frying oil samples.

**Figure 1:**
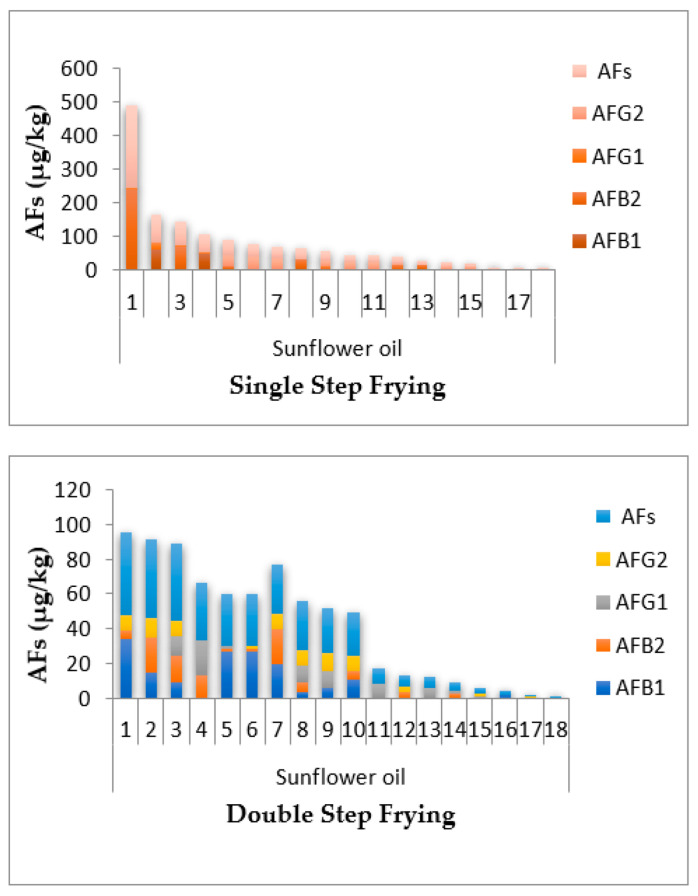
Incidence of total aflatoxins (AFs), aflatoxin G_2_ (AFG_2_), aflatoxin G_1_ (AFG_1_), aflatoxin B_2_ (AFB_2_), and Aflatoxin B_1_ (AFB_1_) in the single and double step frying sunflower oil obtained from Faisalabad, Pakistan.

**Figure 2:**
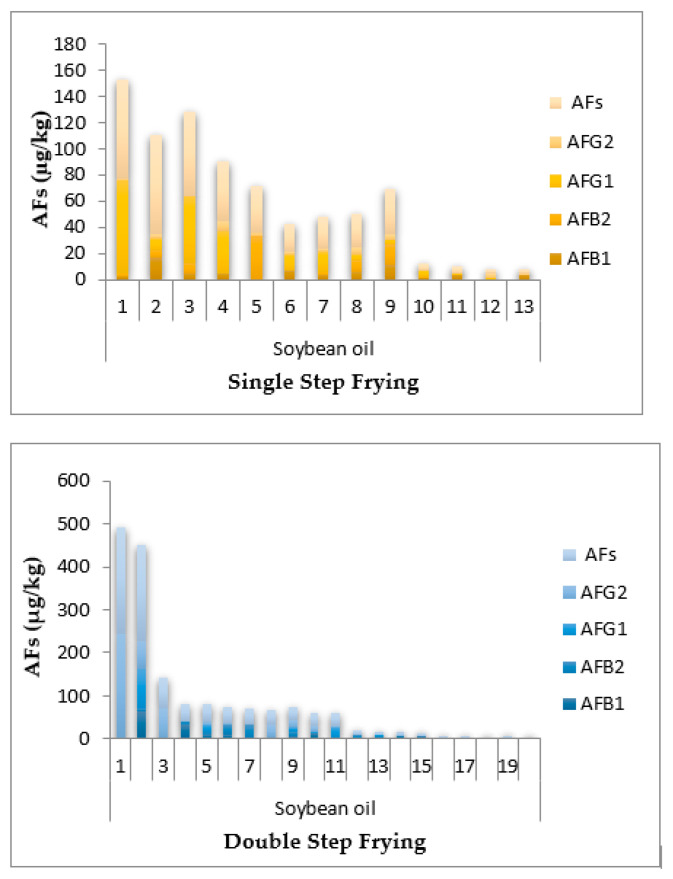
Incidence of total aflatoxins (AFs), aflatoxin G_2_ (AFG_2_), aflatoxin G_1_ (AFG_1_), aflatoxin B_2_ (AFB_2_), and aflatoxin B_1_ (AFB_1_) in the single and double step frying soybean oil samples collected from Faisalabad, Pakistan.

**Figure 3:**
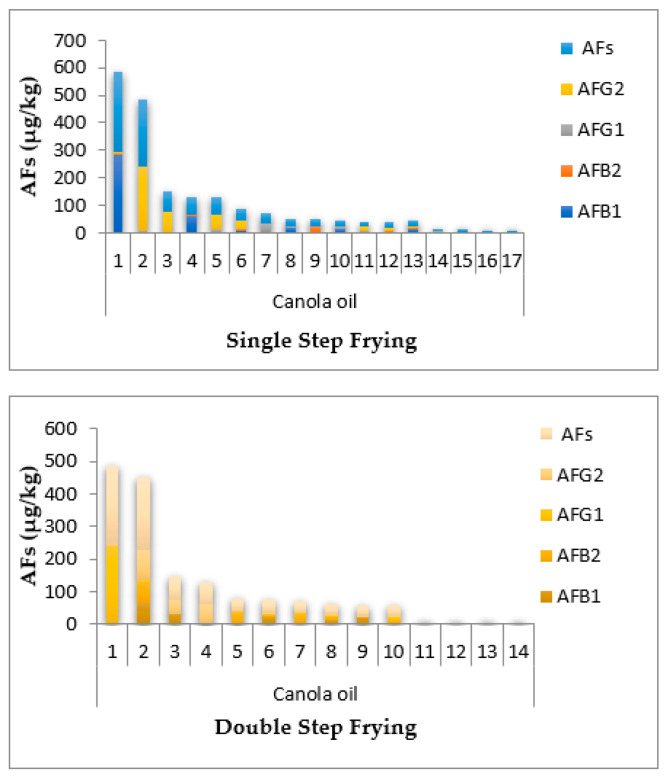
Contamination of total aflatoxins (AFs), aflatoxin G_2_ (AFG_2_), aflatoxin G_1_ (AFG_1_), aflatoxin B_2_ (AFB_2_), and aflatoxin B_1_ (AFB_1_) in the single and double step frying canola oil collected from Faisalabad, Pakistan.

**Figure 4:**
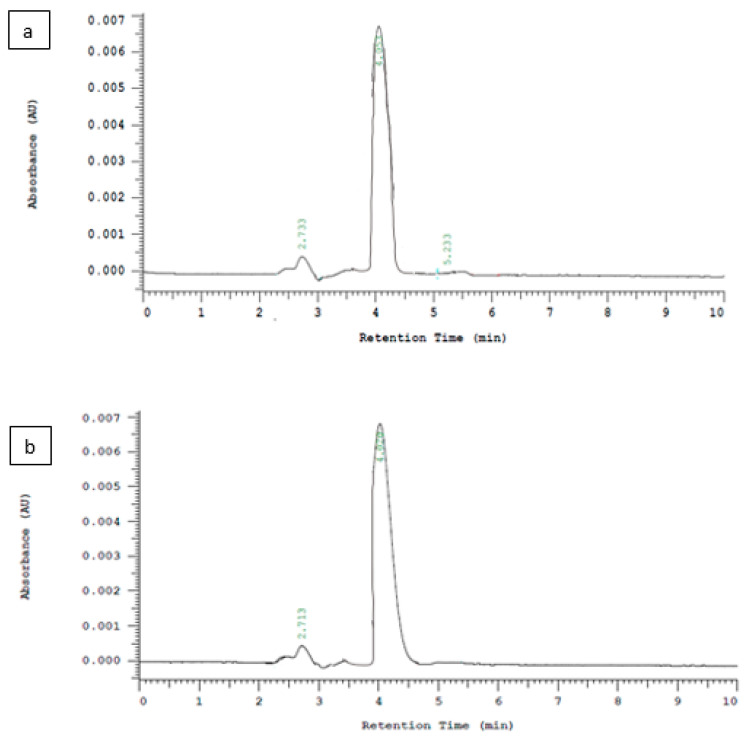

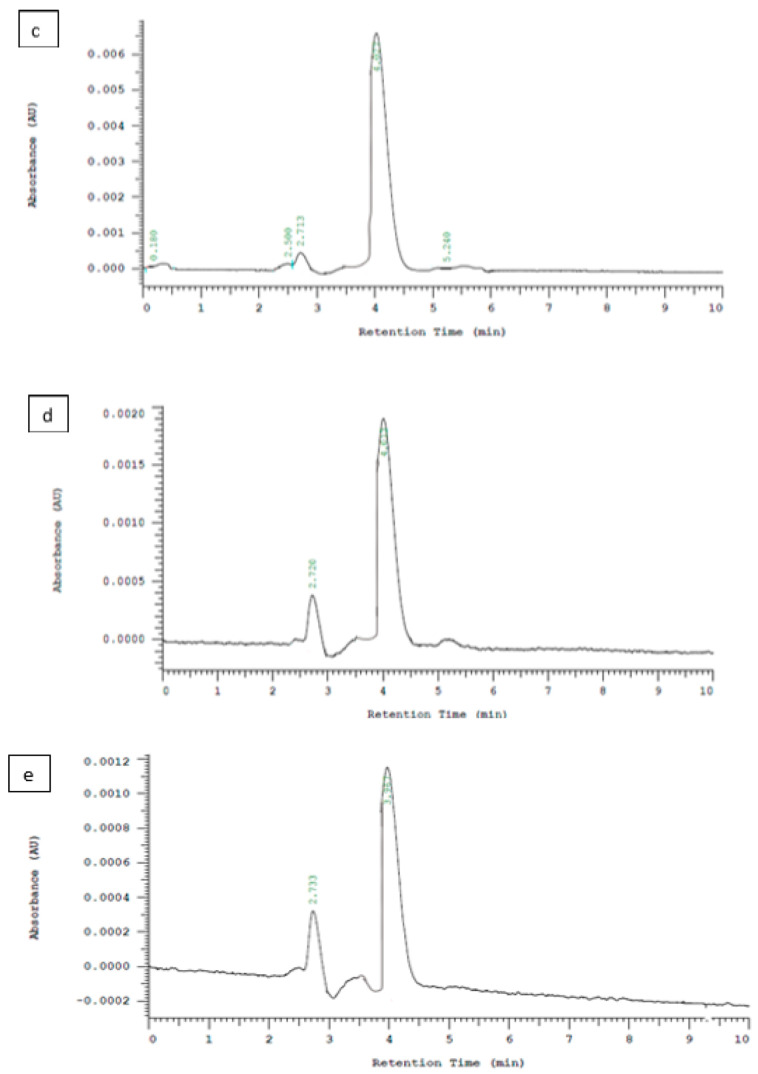
Representative chromatograms of natural incidence of AFB_1_, AFG_1_, AFB_2_, and AFG_2_ in a single frying (**a**) sunflower oil, (**b**) canola oil, and (**c**) soybean oil and double frying (**d**) canola oil and (**e**) soybean oil cooking sample.

Except in the double step frying sunflower oil sample, the highest level of AFB_1_ was in all canola single and double step frying oil samples were above 20 μg/kg, and the lowest observed level of AFB_1_ was in soybean single step frying oil, as shown in Figure 1. Thirty-six sunflower oil samples (single and double step frying oils) had AFB_1_ and AF ranges of 0.025–61.1 and 0.039–231.5 μg/kg, while thirty-two soybean oil samples (single and double oils) had ranges of AFB_1_ and AFs of 0.002–64.2 and 0.006–141.3 μg/kg, respectively, as shown in Figure 2. In addition, 31 samples of canola oil (single and double oils) had AFB_1_ and AFs concentrations ranging from 0.003–72.5 and 0.007–281.1 μg/kg, respectively, as shown in Figure 3. The HPLC chromatograms are shown in Figure 4 for AFTs.

Previous reported work showed that Beheshti and Asadi [23] observed a lower frequency and level of AFs in Iran than the current report’s results. They detected AFB_1_ contamination in 111 (64%) of 173 sunflower samples, with 103 samples (83.7%) of safflower seeds with an average of 2.81 to 0.44 ng/g and 8 samples (16%) of sunflowers with an average of 40.68 ng/g. Only five and two samples of sunflower and safflower seed, correspondingly, exceeded the EU limit (20 µg/kg). Similarly, Karunarathna et al. [11] studied 59 edible oil samples (43 imported and 16 native) from the same geographical region in Sri Lanka (sunflower, sesame, coconut, corn, olive, palm, and soybean oil). They concluded that 12 (37.5 percent) of the 32 coconut samples were infected with aflatoxins. They reported that AFB_1_ and total AF levels ranged from 2.25 to 72.70 µg/kg and 1.76 to 60.92 µg/kg, respectively, with two out of twelve oil samples exceeding the EU’s high permissible limit of 2 µg/kg for AFB_1_. Mohammed et al. [24] investigated 40 samples of sunflower seeds and crude sunflower oil samples (n = 21) from Tanzania and discovered that 6 (15%) samples were contaminated with AFB_1_, ranging from the limit of detection to 218 ng/g, which is lower than the results of the current investigation. Three samples exceed the acceptable limits set by the Tanzanian Bureau of Standards (TBS) and the European Commission/European Union (EC/EU).

Banu and Muthumary [25] found AFB_1_ contamination in 10 (43.4%) of 23 sunflower oil samples from India, and entirely refined samples had levels below the LOD. Mariod and Idris [26] identified AFB_1_ contamination in 54.8 percent of groundnut samples and 14.5 percent of 8 samples of sunflower oil from Sudan. Nabizadeh et al. [27] analyzed 97 edible oil samples from 6 different types (canola, olive, sunflower, and blend, frying, and crude olive oil). They reported that AFB_1_ levels were lower than the LOD in 98 percent of samples and that all positive samples for aflatoxins were below the EU’s 20 µg/kg limit.

In many places around the world, edible oil is regarded as a lucrative cash crop. Nevertheless, the safety and excellence of crops are questioned owing to the use of outdated traditional agricultural methods and inadequate knowledge among traders and farmers regarding toxic fungi. Edible oils (coconut, sunflower, and olive) are frequently stored in cotton sacks for extended periods of time under situations such as contact with the ground, wetness, and temperature extremes. The prolonged period encourages the growth of molds, which favors the colonization of toxicogenic molds [28]. Droughts may impact agriculture during the preharvest period, resulting in the growth of fungi such as *Aspergillus flavus*. To avoid aflatoxigenic fungi growth, extreme temperature and humidity must be regulated, which is a serious concern in humid countries like Pakistan [29]. Additionally, the invasion of AFs in food varies depending on a number of parameters, including the analytical methodologies utilized, ambient conditions, crops, and harvesting practices. The aflatoxigenic fungi can be reduced in food and food products by following excellent postharvest management practices at the level of transportation, processing, packaging, and storage techniques.

### 2.2. Risk Assessment for Health

Several probabilistic studies regarding public health and water and food sources containing various hazards, such as pesticide residues in edible oils [30,31] and polycyclic aromatic hydrocarbons (PAHs) in edible oils, have been performed in recent years [32,33].

Since AFB_1_ was identified in frying cooking oil samples from Faisalabad, Pakistan, a cancer-causing risk evaluation was carried out based on a per capita ingestion of edible cooking oils used in food products, as well as AFB_1_ contamination from the consumed edible oils. A comparison of the levels of contaminants with the accepted limits is insufficient to identify the health state of the exposed population; thus, a health risk assessment should be conducted.

### 2.3. Dietary AFB_1_ Exposure

Table 2 illustrates the estimated daily intake of edible frying oils (sunflower, canola, and soybean oil) used in various fast/fried food items in various age groups of male and female populations from different places in Faisalabad (Pakistan). The most commonly used edible cooking oils in Pakistan were canola, sunflower, and soybean oils, therefore estimating dietary intake from these oils was the most realistic procedure. Local canola oil samples had the highest dietary intake (255.6 and 163.2 µg/kg/day) in male and female adults (over 35 years old), while sunflower oil (100 and 64 µg/kg/day) and soybean oil (78.8 and 50.3 µg/kg/day) samples had lower consumption in male and female adults (over 35 years old) than the canola oil samples. According to the findings, the highest food consumption values were seen in both male and female adults over 35 years of age. Local samples of canola oil revealed the highest levels of dietary intake in both male and female participants as compared to the other oils (sunflower and soybean oils). There were no comparable data available to relate with the results of the present study. Because the country previously lacked adequate health facilities, high AFB_1_ dietary intake levels from canola, soybean, and sunflower consumption would have severe consequences for consumers’ health. Nonetheless, overlooking the individuals’ dieting patterns, seasons, and conventional dietary habits may impact the outcome of dietary intake evaluations.

### 2.4. Risk Characterization Using Margin of Exposure (MOE) Approach

Table 3 shows that the margin of exposure (MOE) values for AFB_1_ exposure from frying cooking oil consumption are based on EDI values calculated from consumption data that were collected across Faisalabad, a city in Punjab Province, using the level of AFB_1_ identified in edible cooking oils (canola, sunflower, and soybean oil) used in fried foods. In comparison to other oils (soybean and sunflower oils), the value of MOE based on human BMDL10 in canola oil samples was substantially lower than 10. The MOE values for consumption of fried oil items from Faisalabad (Pakistan) in canola oil samples were lower than the safe margin of 10,000, as indicated in Table 3. The results show that a number of MOE values were below 10,000, indicating that risk management is possible. Furthermore, the European Commission’s Scientific Panel stated that no findings had been reached regarding human information concerning MOE levels that would be deemed as being of low public health concern. According to a study conducted by the EFS in 2020, predominantly for the younger respondents, the calculated MOEs for all five AFs including AFB_1_ and AFM_1_ were found to be 10,000. This poses an alarming health risk. The assessed cancer perils in humans subsequent to exposure to aflatoxin M_1_ (AFM_1_) and aflatoxin B_1_ (AFB_1_) are in-line with the deduction drawn from the MOEs (EFS, 2020) [34]. No comparable data have been reported to corroborate our conclusion as far as prevalence of food and feed products of Pakistan are concerned. As a result, the BMD and MOE approaches clearly highlighted the need for Pakistan’s risk managers to take action to avoid the negative impact of AFs on consumer health, since these are highly toxic.

**Table 3:** An overview of contact assessment and risk characterization for aflatoxin in various food products in different countries using occurrence.

Sr. No.	Country	Food Item	Contamination (ng/g)	Consumption (g Food Day^−1^)	Exposure (ng/kg Body Weight Day^−1^)	PRPLC *	Ref.
1.	Tanzania	Beer	23	1048	402	33.1	[35]
2.	Ghana	Kenkey	51	1000	850	70.1	[36]
3.	Botswana	Peanut butter	23	20	23	1.9	[37]
4.	Kenya	Maize(commercial)	20	400	133	11	[38]
5.	Gambia	Maize	9.7	22	3.6	0.3	[35]
6.	Kenya	Maize (rural market)	53	400	353	29.2	[39]
7.	Gambia	Groundnut	15	65	16	1.3	[40]
8.	Brazil	Maize	-	-	3.0–17.1	0.057–0.467	[41]
9.	Brazil	Brazil nuts	-	-	6.6–6.8	0.0731–0.0753	[42]
10.	China	Maize	-	-	0.11–5.8	0.003–0.2	[43]
11.	Iran	Bread and peanuts	-	-	3.6	-	[44]
12.	New Zealand	Dried fruits and maize	-	-	0.09	0.0015–0.0019	[45]
13.	Tanzania	Unrefined sunflower oil	0.23	-	-	-	[24]
14.	Iran	Sunflower oil	0.12	-	-	-	[23]
15.	Sudan	Sunflower oil	52.3	-	-	-	[46]
16.	Sudan	Sesame oil	187.6	-	-	-	[46]
17.	China	Oil products	35.0	-	-	-	[47]

* Population Risk for Primary Liver Cancer (Cancers/Year Per 100,000).

### 2.5. Risk Characterization Using Quantitative Liver Cancer Approach 

Table 3 shows an approximation of the population risk (represented as cancers per million) for liver cancer based on a prevalence rate of hepatitis B of 21% acquired from the DHQ hospital in Faisalabad, Pakistan. AFB_1_-related cancer incidence was assessed using 0.0709 cancers per 100,000 people. Table 3 shows that, in a number of circumstances, relatively substantial population hazards are associated with AFB_1_ exposure. Risk managers should consider taking action in a variety of circumstances based on the levels among the demographic groups most vulnerable to exposure. Overall liver cancer occurrence and mortality in Punjab varied greatly among different age groups, with high rates in canola oil (17.13 per 100,000 males over 35 years and 10.93 per 100,000 females over 35 years) and low rates in soybean oil (5.28 per 100,000 males >35 years and 3.37 per 100,000 females over 35 years). According to our conclusions, a quantitative liver cancer approach exposed those males and females of the over 35 age group are at a significant risk of liver cancer in canola oil samples compared to other oils (sunflower and soybean oils). There were no previous data that could be used to compare with this study.

## 3. Conclusions

The present study presents the very first reports of the surveillance of AF contamination in edible oils from Faisalabad, Punjab, Pakistan, to evaluate the risk of liver cancer caused by the exposure of AFB_1_ from consumed edible oil. Furthermore, using a quantitative liver cancer approach and margin of exposure, the cancer-causing risk of AFs from edible oils (single or double stage frying) used in fried food was assessed in males and females of various ages. AF contamination was identified in over 71% (ranges 0.007–281.1 µg/kg) of canola oil samples, some which were above the European regulation’s (20 g/kg) limit. Furthermore, according to the health risk evaluation using the margin of exposure approach (MOE) and the quantitative liver cancer approach, males and females above the age of 35 are at a higher risk of liver cancer as a result of AFB_1_ owing to higher canola oil consumption than other oils. Overall, the assessment highlights the need for AFB_1_ risk assessment throughout Punjab. By using a quantitative liver cancer approach, health risk evaluation stated that males and females over 35 are at considerable risk of liver cancer. Frequent sampling of feed and food samples, as well as training for farmers, merchants, and exporters, could assist in creating awareness of AFs’ toxicity. Additionally, the Punjab Food Authority has undertaken checking food safety and quality, which is a positive step ahead.

## 4. Materials and Methods

### 4.1. Chemicals and Reagents

AF (AFB_1_, AFB_2_, AFG_1_, and AFG_2_) standards (HPLC grade) were obtained from Sigma-Aldrich Co. (St. Louis, MO, USA). Reagents and chemicals obtained included the following: methanol (PubChem CID:34860; ≥99.9%) and acetonitrile (PubChem CID:34998; ≥99.9%), Trifluoroacetic acid (PubChem CID: 302031; ≥99.0%), acetic acid (PubChem CID:695092; ≥99.7%), sodium chloride (NaCl) (Sigma–Aldrich, St. Louis, MO, USA) and moreover, deionized water (PubChem CID: 962) was used in every step of the experiment. Mycosep 226 (AflaZon+) immunoaffinity columns were purchased from Romers Labs (Union, MO, USA).

### 4.2. Sampling

From January 2019 to March 2020, a hundred single and double step frying oil (sunflower, soybean, and canola oil) samples were collected from different local restaurants in Faisalabad (Pakistan). There were three different types of oils, in which sunflower oil samples (n = 36) were obtained from four different brands, canola oil samples (n = 31) were obtained from five different brands, and (n = 33) of soybean oil samples were obtained from six different brands. Each sample of frying cooking oil was picked manually from various restaurants at random. The sample size was kept at 0.5 kg each.

### 4.3. Screening of Cooking Oils for AF Contamination

#### Extraction of AFs from Frying Cooking Oil Samples

The method outlined by Karunarathna et al. [11] for extraction of AFs from frying cooking oils was modified: 25 mL of sample was combined with 125 mL of water:methanol: (45:55 *v*/*v*) and 5 g NaCl. In glass flasks covered with aluminum foil, the mixture was blended at 200 rpm. The Buchner funnel was used to filter the mixture, 70 µL of acetic acid was added to each 9 mL of extract. These acidified extracts were passed through MycoSep 226 immunoaffinity columns (IAC) at a flow rate of around 1 mL/min. Prior to analysis, the obtained AF elute evaporated and was stored for pre-column derivatization. Dried AF residues were mixed with 100 µL trifluoroacetic acid (TFA). For further HPLC analysis, the solutions were kept at room temperature for 20 min in the dark.

### 4.4. HPLC Analysis

For qualitative and quantitative estimation of aflatoxins, all analyses were achieved on an LC-system with the following specifications: HPLC apparatus (Prominance^TM^, shimadzu^®^, Kyoto, Japan) containing a Shimadzu LC software package designed for HPLC real-time and post-operative analysis operated through a computer equipped with Mediterranean Sea 18^®^ 5 μm 25 cm^x^ 0.46 Serial No. N45074 (teknokroma, Sant Cugat del Vallès, Spain) fitted with a CTO-20A^®^ (Shimadzu, Japan) column oven and an LC-20AT^®^ (Shimadzu, Japan) pump. The isocratic mobile phase (20:20:60) of acetonitrile, methanol, and water was used. Flow rate was kept at 1.5 mL/min. Injection volume was 20 µL: Rheodyne^®^ sample injector with 20 μL sample loop. The elute was detected using a spectrofluorometer detector RF-10AXL ^®^ (shimadzu, Japan) set at emission 440 nm and excitation at 360 nm.

### 4.5. Parameters for Method Validation in AF Analysis

Over the entire range of AFs injected, the standard calibration curves were linear. The concentration range for AFG_1_ was determined to be 0.005–20 ng mL^−1^, for AFB_1_ 0.005–150 ng mL^−1^, for AFB_2_ 0.002–20 ng mL^−1^, and for AFG_2_ 0.002–6.0 ng mL^−1^. Limit of detection (LOD) was calculated using a signal to noise ratio (S/N) of 3 and limit of quantification (LOQ) was calculated using a signal to noise ratio (S/N) of 10. In the Table 4, the correlation coefficients are presented.

### 4.6. Online Survey from Different Places in Faisalabad

**Figure 5:**
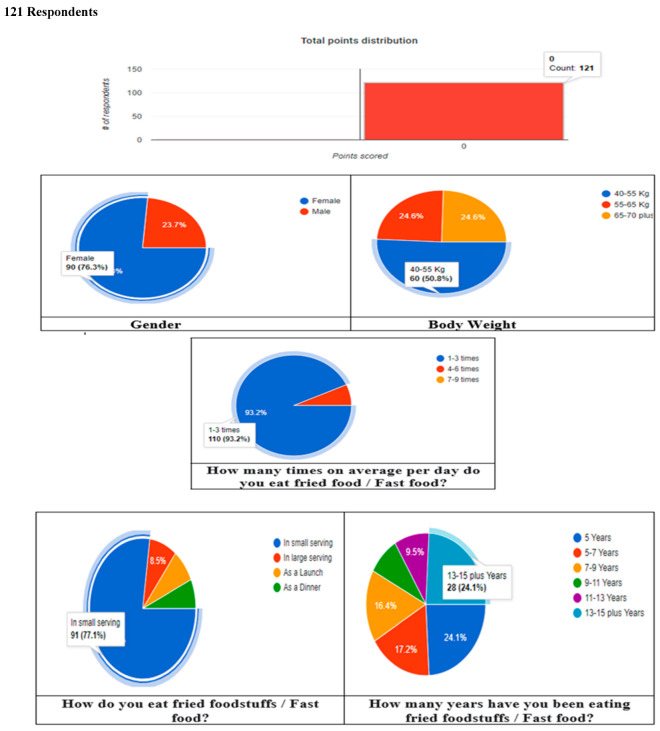
Responses to questionnaire used in survey.

A survey using a questionnaire was conducted to estimate the consumption data of edible cooking oils (single and double frying) used in fried foods and fast foods for approximately 121 participants and to ask them about the fried food oils consumed in the previous two years. The participants’ average weight was 55 kg. The survey took into account all factors of oil consumption, including food additives and consistency Figure 5. These studies determined the exact amount of edible cooking oil used in food products (both single and double frying). https://docs.google.com/forms/d/1NvbQU_khyhnMMgRFW7Q9g1yhj-62uoPPub5lH3vmMw0/edit. Accessed on 12 September 2020.

### 4.7. Estimation of Daily Food Intake

Equation (1) was used to analyze the estimated daily intake (EDI) provided by [48].

(1) $$\mathrm{Dietary}\;\mathrm{food}\;\mathrm{intake}\;\left(\mathrm{µg}/\mathrm{kg}/\mathrm{day}\right)=\overset{n}{\underset{i=1}{\sum }}=\frac{\mathrm{Ci}\;\times \;\mathrm{Di}}{\mathrm{BW}}$$

Ci represents AFB_1_ concentration (µg/kg), Di represents dietary intake of edible cooking oil (single and double frying) used in fried foods and fast foods (g/person/day), and BW depicts mean body weight (kg).

### 4.8. Risk Assessment

Two different methods have been proposed to assess risk. International regulatory agencies have employed the EFSA’s margin of exposure (MOE) approach [48] as well as the FAO and WHO’s quantitative liver cancer risk approach [49].

#### 4.8.1. MOE (Margin of Exposure) Approach

The EFSA systematized the approach to the margin of exposure (MOE) to evaluate the threat of a genotoxic and carcinogenic material [50]. The MOE is not used to measure the safety of regulated substances consciously supplemented in the food chain but is a proportion of two aspects which estimates for a specific population the dose at which a modest but quantifiable adverse effect is first noticed at the level of exposure to the element under consideration. The EFSA Scientific Panel on Contaminants in the Food Chain advocated describing the MOE using the benchmark dose lower confidence limit for 10% excess risk (BMDL10) [51]. The EC Scientific Panel [52] established BMDL10 for a 10% enhanced cancer risk using human data (870 ng/kg body weight/day) for the risk valuation of AFB_1_. The MOE is the combination of the BMDL10 and the EDI. If the BMDL10 is employed as the MOE calculation parameter, the EDI is regarded to be of public health concern and the MOE value will be less than 10,000 [53]. It should be noted that MOE values do not quantify danger, but rather these suggest a level of concern: the lower the margin of exposure value, the greater the level of concern [54].

#### 4.8.2. Quantitative Liver Cancer Risk Approach

The FAO and WHO have embarked on a project to quantitatively assess the risk of liver cancer induced by AFB_1_ exposure [49]. The carcinogenic potency (represented in the range of cancers/year/100,000 populations/ngAFB_1_/kg/day) is multiplied by the total consumption of AFB_1_ (articulated in ng AFB_1_/kg/day) [55]. Actually, the carcinogenic potency was studied in both people with hepatitis B (using the HBsAg+) and persons who did not have hepatitis B (using the HBsAg−). Hepatitis B is known to enhance the risk of AFB_1_-induced liver cancer in a synergistic manner [56]. In the current study, the prevalence rate of hepatitis B positive patients from Faisalabad (Pakistan) was 21 percent, which was used to estimate the risk.

Cancer risk was determined by using Equation (2) [22].

(2)  Average Potency=(PHBsAg+× pop.HBsAg+)+(PHBsAg−× pop.HBsAg−)Cancer risk = Average Potency × EDI (Estimated Daily Intake)

The prevalence rate of hepatitis B positive patients associated with gender and age group was obtained. These data aimed to evaluate the HBV occurrence rate and its risk factors among the general population visiting the DHQ hospital of Faisalabad, Pakistan.

### 4.9. Statistical Analysis

The current study was statistically analyzed, and the consequences were described as mean ± standard deviations. The results of an analysis of variance of data on aflatoxins B_1_, B_2_, G_1_, and G_2_ in single and double frying oils from sunflower, canola, and soybean oils show that the effect of treatment interaction between oil types (sunflower, soybean, and canola oils) and single and double frying oils for aflatoxins B_1,_ B_2_, G_1_, and G_2_ was no significant difference (D. F = 3, *p* = 0.003, Fcal = 11.22 value).

## Figures and Tables

**Table 1:** Level of AFB_1_, AFB2, AFG_1_, and AFG_2_ with total AFs in canola oils, sunflower oils, and soybean oils from different places of Faisalabad, Pakistan.

Edible Oils	Step	N	Positive Samples N (%)	Below Permissible Limit <20 µg kg^−1^	Above Permissible Limit >20 µg kg^−1^	AFB_1_ µg/kg (Mean ± SD)	AFB_2_ µg/kg (Mean ± SD)	AFG_1_ µg/kg (Mean ± SD)	AFG_2_ µg/kg (Mean ± SD)	AFs µg/kg (Mean ± SD)	Ranges (µg/kg)	
											AFB_1_	AFs
**Canola** **oil**	**1 Step** **Frying**	17	17 (100)	4	13	24.64 ± 0.82	6.90 ± 0.74	3.80 ± 0.67	52.16 ± 0.52	87.51 ± 0.91	0.003–72.5	0.007–281.1
	**2 Step** **Frying**	14	10 (7142)	4	10	11.98 ± 0.93	26.96 ± 0.58	30.13 ± 0.89	15.33 ± 0.65	82.93 ± 0.88	0.012–62.4	0.016–241.5
**Sunflower** **oil**	**1 Step** **Frying**	18	12 (66.66)	6	12	6.76 ± 0.69	41.98 ± 0.96	3.74 ± 0.79	19.9 ± 0.81	72.38 ± 0.95	0.025–61.1	0.039–231.5
	**2 Step** **Frying**	18	10 (55.55)	8	10	8.56 ± 0.68	10.24 ± 0.91	6.73 ± 0.66	8.97 ± 0.78	34.5 ± 0.82	0.045–27.1	0.071–54.4
**Soybean** **oil**	**1 Step** **Frying**	13	9 (69.23)	4	9	5 ± 0.71	7.96 ± 0.56	22.61 ± 0.90	7.87 ± 0.62	43.44 ± 0.68	0.038–16.6	0.088–71.2
	**2 Step** **Frying**	20	11 (57.89)	9	11	8.65 ± 0.61	13.39 ± 0.82	13.64 ± 0.71	39.98 ± 0.53	5.66 ± 0.97	0.002–64.2	0.006–141.3
**Total**		100	89 (89.00)	35	65							

**Table 2:** Risk characterization on margin of exposure (MOE) and population risk approach for canola oil, soybean oil, and sunflower oil (based on a model formulated by EFSA 2005–2007). Exposure evaluation had an average body weight of 55 kg. Population risk is estimated from the prevalence rate of 21% HBsAg.

Edible Cooking Oil (Frying)	Type	Male			Female		
		Age Group			Age Group		
		≤22	≤27	>35	≤22	≤27	>35
**Canola**	**Contamination (µg/g)**	18.34	18.34	18.34	18.34	18.34	18.34
	**Consumption (g/day)**	226	355	885	142	354	565
	**Exposure (ng/kg/day)**	65.29	102.56	255.68	41.02	102.2	163.23
	MOE for human * BMDL10	13.32	8.47	3.40	21.20	8.51	5.32
	Population risk * **(cancers/year/100,000)**	4.37	6.87	17.13	2.74	6.85	10.93
**Soybean**	**Contamination (µg/g)**	6.96	6.96	6.96	6.96	6.96	6.96
	**Consumption (g/day)**	226	355	885	142	354	565
	**Exposure(ng/kg/day)**	20.13	31.62	78.84	12.65	31.53	50.33
	MOE for human * BMDL10	43.21	27.51	11.03	68.77	27.59	17.28
	Population risk * **(cancers/year/100,000)**	1.34	2.11	5.28	0.84	2.11	3.37
**Sunflower**	**Contamination (µg/g)**	7.66	7.66	7.66	7.66	7.66	7.66
	**Consumption (g/day)**	226	355	885	142	354	565
	**Exposure (ng/kg/day)**	25.64	40.27	100	16.11	40.16	64.14
	MOE for human * BMDL10	33.93	21.60	8.66	54	21.66	13.56
	Population risk * **(cancers/year/100,000)**	1.71	2.69	6.72	1.07	2.69	4.29

* Benchmark dose (BMD) lower limit for 10% extra risk, taken as 870 ng/kg/day. * Population risk for primary liver cancer (cancers/year/100,000 persons).

**Table 4:** Method validation for HPLC analysis for the detection of AFs.

Aflatoxin	Rt (min)	LOD * (ng mL^−1^)	LOQ * (ng mL^−1^)	Calibration Curve	R^2^
**AFB_1_**	5.24	0.002	0.005	y = 67093x + 33.842	0.9997
**AFB_2_**	4.02	0.001	0.002	y = 45747x − 709.54	0.9995
**AFG_1_**	2.71	0.002	0.005	y = 33025x + 27.800	0.9996
**AFG_2_**	2.50	0.001	0.002	y = 63802x − 85.618	0.9991

LOD * = limit of detection. LOQ * = limits of quantification. Rt = retention time.

## Data Availability

The data presented in this study are available in this article.
